# *Agastache* Species: A Comprehensive Review on Phytochemical Composition and Therapeutic Properties

**DOI:** 10.3390/plants12162937

**Published:** 2023-08-14

**Authors:** Mihaela-Ancuța Nechita, Anca Toiu, Daniela Benedec, Daniela Hanganu, Irina Ielciu, Ovidiu Oniga, Vlad-Ionuț Nechita, Ilioara Oniga

**Affiliations:** 1 Department of Pharmacognosy, Faculty of Pharmacy, “Iuliu Hațieganu” University of Medicine and Pharmacy, 12 Ion Creangă Street, 400010 Cluj-Napoca, Romania; 2 Department of Pharmaceutical Botany, Faculty of Pharmacy, “Iuliu Haţieganu” University of Medicine and Pharmacy, 23 Gheorghe Marinescu Street, 400337 Cluj-Napoca, Romania; 3 Department of Pharmaceutical Chemistry, Faculty of Pharmacy, “Iuliu Hațieganu” University of Medicine and Pharmacy, 41 Victor Babeș Street, 400010 Cluj-Napoca, Romania; 4 Department of Medical Informatics and Biostatistics, Faculty of Medicine, “Iuliu Hațieganu” University of Medicine and Pharmacy, 6 Louis Pasteur Street, 400349 Cluj-Napoca, Romania

**Keywords:** *Agastache* species, phytochemistry, bioactivity

## Abstract

The *Agastache* genus is part of the *Lamiaceae* family and is native to North America, while one species, *Agastache rugosa* (*A. rugosa*), is native to East Asia. A review on the phytochemistry and bioactivity of *Agastache* genus was last performed in 2014. Since then, a lot of progress has been made on the characterization of the phytochemical and pharmacological profiles of *Agastache* species. Thus, the purpose of this paper is to present a summary of the findings on the phytochemistry and biological effects of several *Agastache* species, including both extracts and essential oil characterization. We performed a comprehensive search using PubMed and Scopus databases, following PRISMA criteria regarding the study selection process. The available data is focused mainly on the description of the chemical composition and bioactivity of *A. rugosa*, with fewer reports referring to *Agastache mexicana* (*A. mexicana*) and *Agastache foeniculum* (*A. foeniculum*). *Agastache* species are characterized by the dominance of flavonoids and phenolic acids, as well as volatile compounds, particularly phenylpropanoids and monoterpenes. Moreover, a series of pharmacological effects, including antioxidant, cytotoxic, antimicrobial, anti-atherosclerotic, and cardioprotective properties, have been reported for species from the *Agastache* genus.

## 1. Introduction

*Agastache* Clayton ex Gronov is a small genus of the *Lamiaceae* family that consists of 22 species known under the common name ‘giant hyssop’. *Agastache* species (sp.) are native to North America, with one species originating from East Asia. *Agastache* sp. is used as a medicinal and ornamental plant and is known in traditional medicine as a natural remedy against pain, hypertension, and gastrointestinal diseases [1,2,3,4].

Overall, the *Agastache* genus appears to be less investigated compared with other genera, both from a phytochemical and therapeutic perspective. The chemical composition and pharmacological profile of some *Agastache* sp. have not been described yet, and the available data is limited compared with other medicinal plants.

This review includes information mainly on the following species: *A. rugosa* (Fisch. & C.A. Mey.) Kuntze; *A. foeniculum* (Pursh) Kuntze; and *A. mexicana* (Kunth) Lint & Epling. Other species are also mentioned, but there is less data available in the literature. Most studies refer to the composition and bioactivity of *A. rugosa* extracts and essential oils. Known under the popular name of Korean mint, *A. rugosa* is native to East Asian countries, including China, Japan, and Korea. *A. rugosa* has empirically been used to treat cholera, vomiting, miasma, and other gastrointestinal issues [2,5,6].

The last review in English on the *Agastache* genus was published in 2014 by Zielińska et al. [7]. Since then, there has been significant progress on the pharmacognostic characterization of *Agastache* sp. Also, the acclimatization of *Agastache* sp. in Romania raised our interest in a better understanding of the phytochemistry and biological effects of the extracts and essential oils of *Agastache* species. Thus, the aim of this paper was to provide a comprehensive review of the *Agastache* genus, including information regarding its chemical composition and bioactivity.

## 2. Methodology

We performed a literature review following the Preferred Reporting Items for Systematic Reviews and Meta-Analyses (PRISMA) criteria [8], including references published up to February 2023 in PubMed and Scopus databases. We used a combination of keywords and MeSH terminology to perform a comprehensive review of the literature. The following formula was used in the PubMed search: (“Agastache” [Mesh] OR Agastache) AND (“chemistry” [Subheading] OR “Pharmacologic Actions” [Mesh] OR phytochemistry OR “chemical composition” OR compound* OR component* OR metabolite* OR bioactivity OR “biological action*” OR “pharmacological effect*” OR “pharmacological action*” OR pharmacology OR bioactive). When searching in Scopus, the following searching strategy was used: (TITLE-ABS-KEY (agastache)) AND ((TITLE-ABS-KEY (pharmacolog* OR bioactiv* OR therap*)) OR (TITLE-ABS-KEY (composition OR phytochem* OR compound* OR component* OR metabolite*))).

**Figure 1:**
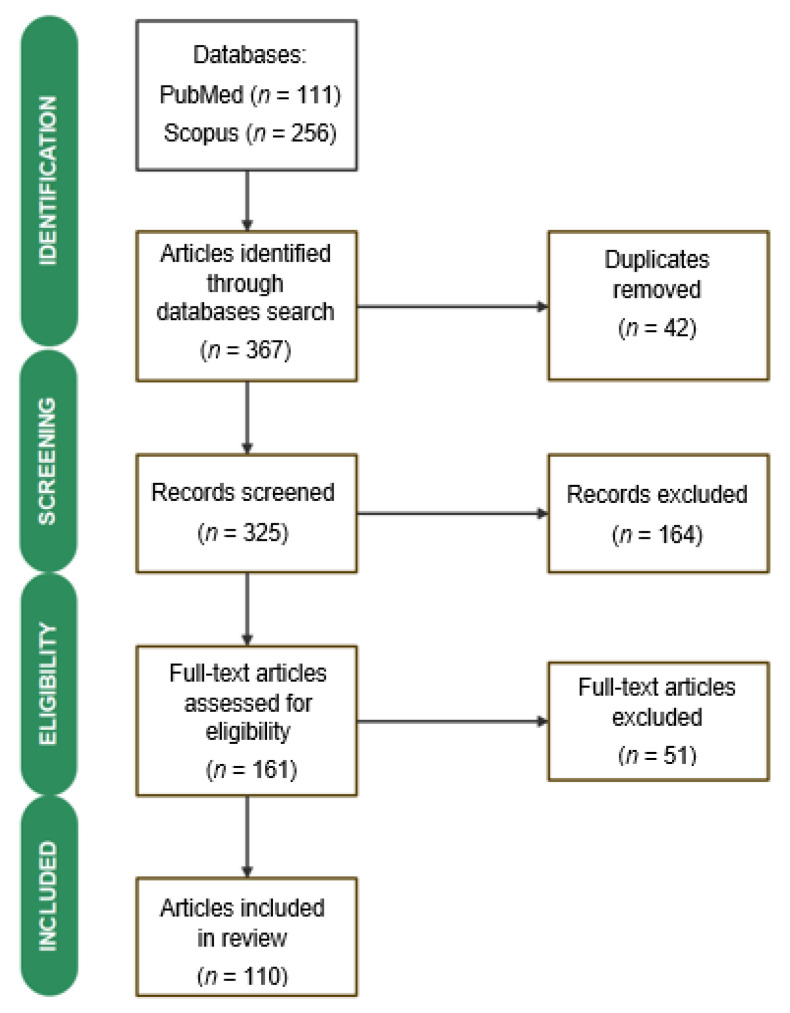
Study selection process.

In total, 367 references were found, of which 111 were on PubMed and 256 on Scopus. After removing duplicates (*n* = 42), the following selection criteria were used: studies published in English, the presence of the keyword *Agastache* in the title and/or abstract, the study topic, excluding studies referring solely to agronomy, as well as the absence of any data on their phytochemistry or pharmacology. Following this preliminary screening, 164 manuscripts were eliminated. The remaining 161 full-text documents were carefully examined. Next, 51 articles were removed, given the lack of information regarding the phytochemical composition or therapeutic properties of *Agastache* species. Finally, this evaluation comprised 110 references. The study selection process is presented in Figure 1.

## 3. Phytochemical Profile

The phytochemical profile of *Agastache* sp. consists mainly of phenolic compounds and terpenoids. The available data describes the phytochemical characterization of both extracts and essential oils from *Agastache* sp. Most of the research has been conducted on essential oils, which contain estragole, limonene, menthone, and pulegone as major compounds, depending on species and chemotype. Additionally, due to their numerous medicinal qualities, the polyphenolic components of *Agastache* sp., such as flavonoids and caffeic acid derivatives, have also been investigated.

### 3.1. Chemical Composition of Agastache *sp.* Extracts

**Figure 2:**
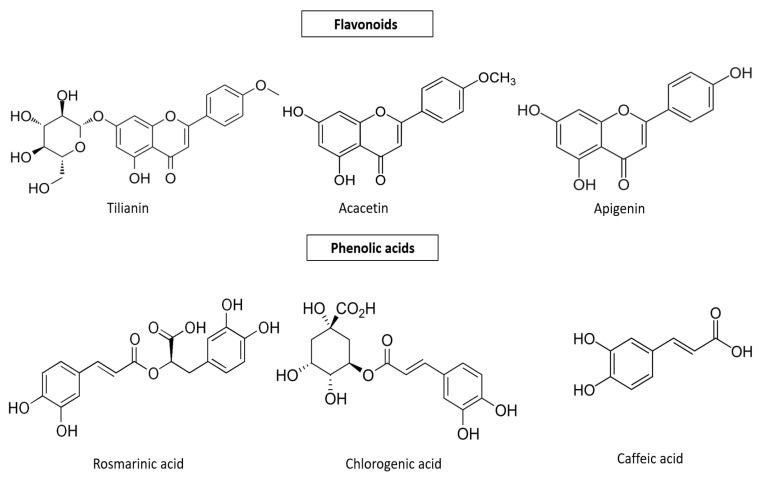
Chemical structures of major polyphenolic compounds of *Agastache* sp. extracts.

The major polyphenolic compounds of *Agastache* sp. extracts are listed in Table 1, and their structures are presented in Figure 2. The extracts were performed using methanol, ethanol, or water as solvents, and the authors identified polyphenolic compounds in flowers, leaves, and stems, while agastaquinone was found in hexane extract from roots.

#### 3.1.1. *Agastache rugosa*

*A. rugosa* extracts contain mainly phenolic acids (including caffeic acid derivatives and hydroxycinnamic acid derivatives) and flavonoid derivatives [9]. Total polyphenolic content for *A. rugosa* ethanolic extract was 38.11 ± 0.88 mg /g extract [17], with similar values for the aqueous extract, 38.9 ± 1.7 mg/g [29]. The total flavonoid (TFC) content in *A. rugosa* aqueous extract was reported to be 22.8 ± 7.6 mg (naringin equivalents/g extract) [30].

Rosmarinic acid was reported to be one of the main components of *A. rugosa* ethanolic [11], aqueous [18], and methanolic extracts [9,31], and it was found in a concentration of 25.81 mg/g in the ethanolic extract [19]. Other phenolic acids identified in *A. rugosa* extracts were chlorogenic acid [9], cryptochlorogenic acid, feruloyl-quinic acid [31], protocatechuic acid [23], salvianolic acid [32], and hydroxycinnamic acid [12].

Another class of major compounds in *A. rugosa* extracts is represented by flavonoid derivatives. Tilianin (acacetin 7-*O*-glucoside) and its aglycon acacetin were reported more frequently [11,23,32,33]. Some authors reported values of 21.14 mg/g tilianin and 9.94 mg/g acacetin [17], while others found a concentration of 38.29 mg/g tilianin and 7.88 mg/g acacetin [19]. Another study also confirmed that tilianin contents were noticeably higher in the leaves and flowers than acacetin concentrations [34].

Pentacyclic triterpenoids (ursolic acid), sterols (β-sitosterol) [23], and carotenoids (lutein, zeaxanthin, β-cryptoxanthin, α-carotene, β-carotene, 13Z-β-carotene, and 9Z-β-carotene) were also identified in *A. rugosa* extracts [22]. Some older studies reported the presence of terpenoid and lignan-type compounds: agastaquinone, a diterpenoid quinone isolated from the roots of *A. rugosa* [20]; agastinol and agastenol, two lignan compounds isolated from the whole plant of *A. rugosa* [35]. We found no recent studies reporting the presence of this type of compound (agastaquinone, agastinol, or agastenol).

The concentration of the active compounds in *A. rugosa* extracts depends on various factors. Some authors investigated the variation of the composition with the age of the plant and found the maximum concentration of active compounds in two-year-old *A. rugosa* (25.81 mg/g d.w. rosmarinic acid, 15.61 mg/g d.w. apigenin glucoside, and 7.24 mg/g d.w. chlorogenic acid) [9]. Zielińska et al. also found that two-year-old *A. rugosa* leaves were richer in rosmarinic and chlorogenic acids than one-year-old ones. In contrast, there was no link between the amount of these compounds in the inflorescences and the age of the plant [10].

The composition of *A. rugosa* extract also depends on the part of the plant used. Thus, Park et al. found that flowers had the highest content of phenolic compounds, flavonoids, and anthocyanins, followed by leaves and stems. In contrast, HPLC analysis showed that the total phenylpropanoid content was highest in the leaves [15]. Desta et al. also confirmed that the highest concentration of flavonoids was found in flowers (89.43%), followed by leaves (89.25%), stems, and roots of *A. rugosa* [12]. The concentration of tilianin in the leaves was 2.18 µg/g, whereas the amount in the flowers was 6.33 µg/g. The stems and roots only had trace amounts of tilianin in them, 0.49 µg/g and 0.14 µg/g, respectively [34].

Yeo et al. showed that the profile of primary and secondary metabolites is also influenced by the period of floral development. The total polyphenolic content of the methanolic extract of *A. rugosa* was maximum during the budding period, stage I (3.96 ± 0.06 mg/g d.w.), while the total flavonoid content was maximum at stage II, the partial flowering stage (0.68 ± 0.01 mg/g d.w.). HPLC analysis showed that 4-hydroxybenzoic acid, chlorogenic acid, *trans*-cinnamic acid, and quercetin levels were highest in stage I, while levels of caffeic acid, (−)-epicatechin, tilianin, and acacetin were maximum in stage II. Rutin and rosmarinic acid levels were highest in stage III, the full flowering stage [16]. Another study found that the content of rosmarinic acid is increasing during the vegetation season, from 0.63 ± 0.04 mg/g d.w. in June to 1.88 ± 0.09 mg/g d.w. in August (one-year-old plants) and from 3.7 ± 0.26 mg/g d.w. in June to 9.31 ± 0.24 mg/g d.w. in August (two-year-old plants) [9]. Other researchers showed that the highest content of tilianin and acacetin was observed in *A. rugosa* collected at 12–13th weeks after seeding. Thus, it is recommended to harvest the plant material 12–13 weeks after seeding due to the high content of active compounds [13].

Regarding the differences in composition depending on the origin of the plant, tilianin and acacetin were reported as the main compounds in *A. rugosa* from China [23,24], while *A. rugosa* from Korea presented a more varied composition, depending on the part of the plant used and type of extract: some reported rosmarinic acid as the major compound [11,12,18], others acacetin 7-*O*-(6″-*O*-malonyl)-β-D-glucopyranoside [13] or tilianin [16,17,19,22]. Regarding *A. rugosa* from Poland, researchers reported rosmarinic acid as one of the predominant compounds [9,10].

#### 3.1.2. *Agastache foeniculum*

Regarding the composition of *A. foeniculum*, phenolic acids (caffeic acid and *p*-coumaric acid) as well as flavonoids (quercetin, genistein, hyperoside, and rutoside) were identified in the extracts. The concentrations of the bioactive compounds were higher in the methanolic extracts compared with the ethanolic extracts. Total polyphenolic content (TPC) was 485.08 mg gallic acid equivalents (GAE)/g for the methanolic extract and 403.91 mg GAE/g for the ethanolic extract [26]. These results are higher than those reported by others, who reported a TPC of 1.14 mg hydroquinone equivalents/g for the aqueous extract [36] and 2.88 mg GAE/g for the ethanolic extract [25]. However, differences regarding the extraction method, part of the plant used, and origin could lead to variations in results. *A. foeniculum* has more flavonoids than phenolic acids, so using gallic acid for the calibration curve is not entirely correct; the optimum choice for a calibration curve is a flavone [27]. *A. foeniculum* harvested at the beginning of flowering and in the afternoon had the highest amounts of polyphenols (27.19 mg GAE/g dry plant). There were no statistically significant variations in polyphenolic content after 10 days (full bloom) [27].

One study showed that the total flavonoid content was 367.32 mg quercetin equivalents (QE)/g for the methanolic extract and 355.94 mg QE/g for the ethanolic extract [26]. Lower values have been reported by others: 31.87 mg QE/g [27], 1.07 mg QE/g [36], and 2.21 mg QE/g [25]. Some authors reported the results in rutin equivalents (RE) and obtained a total flavonoid content of 6.5 mg RE/g [37].

#### 3.1.3. *Agastache mexicana*

*A. mexicana* ssp. *mexicana* and *A. mexicana* ssp. *xolocotziana* displayed a similar chemical profile, with tilianin, acacetin [38], and (2-acetyl)-7-*O*-glucosyl acacetin as the most abundant compounds [39]. These findings are consistent with previous research on other *Agastache* species. Nevertheless, there were significant differences in the relative abundance of their constituents. For example, tilianin and acacetin were more abundant in *A. mexicana* ssp. *xolocotziana* than in ssp. *mexicana* [38]. Another study pointed out that acacetin 7-*O*-(6″-*O*-malonyl)-glucoside was the predominant component of subsp. *mexicana* (20.43 g/g extract), while subsp. *xolocotziana* contains only 1.26 g/g extract. Also, some authors concluded that *A. mexicana* ssp. *mexicana* has a higher total polyphenolic and flavonoid content than ssp. *xolocotziana* [28].

### 3.2. Chemical Composition of Essential Oils from Agastache Species

*Agastache* species are traditionally used as spices due to the characteristic anise-like aroma given by the essential oil. Different factors could determine changes in essential oil composition, such as cultivation methods, origin, age of the plant, weather conditions [40,41], and chemotype differences [42].

**Figure 3:**
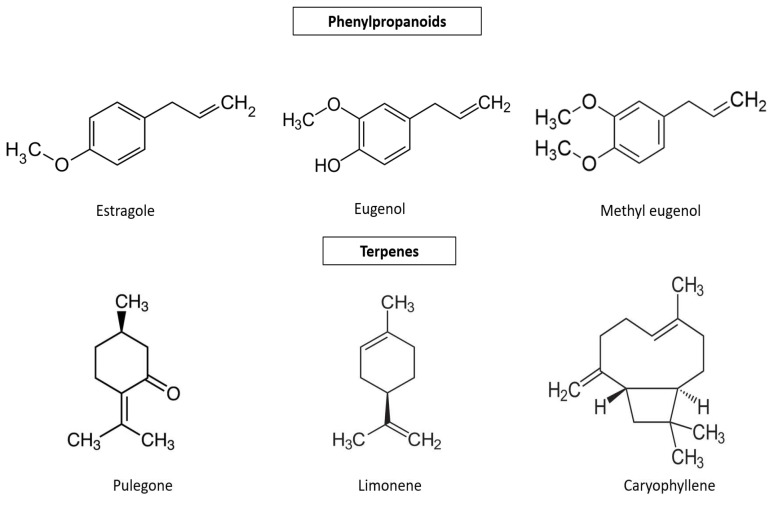
Chemical structures of major volatile compounds of *Agastache* sp. essential oils.

The aromatic profile of *Agastache* sp. is characterized by the presence of monoterpenes, sesquiterpenes, phenylpropanoids, and non-terpene derivatives. Essential oils are characterized by the dominance of phenylpropanoids (estragole = methyl chavicol, methyl eugenol), while others are characterized by the dominance of terpenoid compounds (limonene, menthone, pulegone). The major volatile compounds identified in *Agastache* sp. are listed in Table 2, and their chemical structures are presented in Figure 3.

#### 3.2.1. *Agastache rugosa* Essential Oil

Most studies on *A. rugosa* found that estragole was the major compound of the volatile oil [42,43,45,47,48,62], while others identified methyl eugenol [53], pulegone, *p*-menthan-3-one [50], or limonene [49] as the main components. Other volatile compounds identified in high amounts were linalool [48], β-caryophyllene, humulene, germacrene, bicyclogermacrene [43], anethole, and 2-phenyl propionaldehyde [47].

Yamani et al. showed that estragole was the predominant compound of the Australian-grown *A. rugosa* volatile oil, reaching maximum concentration in nectar (97.16%), followed by flowers (96.74%) and leaves (94.35%) [43]. In contrast, another study found that *A. rugosa* honey contained only 12.31% estragole, suggesting that the nectar contained only a low amount of the compound compared with flowers and leaves [44]. Najafi et al. also identified estragole as the main component of *A. rugosa* volatile oil and as one of the major compounds of the oil fraction, along with linolenic acid, palmitic acid, linoleic acid, and oleic acid [62]. On the contrary, pulegone was the major compound in the volatile oil from the flowers of *A. rugosa* from China, and *p*-menthan-3-one was the most abundant in the volatile oil extracted from leaves [50]. In another study, the main constituents of *A. rugosa* essential oil were methyl eugenol (50.51%), estragole (8.55%), and eugenol (7.54%). Most of the volatile oil (66.60%) was composed of phenylpropanoid compounds, with just 14.22% monoterpenoids and 13.34% sesquiterpenoids [53].

Seven compounds, 2,6-dimethylheptane, 4-methyl-1-pentene-3-ol, δ-limonene, *trans*-ocimene, octenyl acetate, anethole, and 2-methyl-5-isopropenyl-2-cyclohexenone, were present only in the volatile oil from the flowers of *A. rugosa*, while β-damascenone and allyl-3-methyl-2-butanoate were present only in the volatile oil from the leaves [47]. Thus, there are differences in the composition of the volatile oil depending on the location of the plant and the part of the plant used. The concentrations of these volatile compounds fluctuated considerably throughout time, with the largest oil yield occurring at the beginning of the growth season (May) [48].

Given the diversity in the chemical composition of the volatile oil from the same species, *A. rugosa* was divided into several chemotypes. Chae et al. (2005) suggested that there are five major chemotypes: estragole, methyl eugenol, methyl eugenol + limonene, menthone, and menthone + pulegone [63].

Interestingly, other researchers have identified variations in the volatile oil composition in populations with the same cultivation method and location. Thus, among the 90 populations of *A. rugosa*, estragole, D-limonene, and isopulegone were found in the highest concentrations. Depending on the chemical composition of the volatile oil, the populations were divided into six chemotypes: estragole, pulegone, methyl eugenol, menthone, isopulegone, and nepetalactone. Thus, although the cultivated seeds had the same environmental conditions, the composition of the volatile oils varied significantly [46].

Following the same pattern, a comparative analysis performed by Dang et al. showed the differences between two chemotypes of *A. rugosa: the* pulegone chemotype and the estragole chemotype. The volatile oil of the pulegone chemotype was characterized by the dominance of pulegone, isomenthone, and limonene (monoterpenoids). Estragole and methyl eugenol were not found in the pulegone chemotype. In contrast, the major compounds of the volatile oil in the estragole chemotype were estragole and caryophyllene [55].

The chemical profile of the volatile constituents of the suspension culture of *A. rugosa* had major differences compared with the intact plant. In the suspension culture, 3-hydroxi-2-butanone and 2,4,5-trimethyl-3-oxazoline were reported as the major compounds, while estragole, the major compound of the parent plant, was not identified in the suspension culture [64].

#### 3.2.2. *Agastache mexicana* Essential Oil

Regarding *A. mexicana* ssp. *mexicana*, one study highlighted estragole, D-limonene, and linalyl anthranilate as the major compounds of the volatile oil [65]. These results are in accordance with those previously reported by Estrada-Reyes et al., who identified estragole, limonene, and linalool as dominant compounds in *A. mexicana* ssp. *mexicana* [39]. Regarding *A. mexicana* ssp. *xolocotziana*, one study showed the dominance of phenylpropanoids (methyl eugenol and estragole) [66], and another study showed the dominance of terpenes (pulegone, menthone, and isopulegone) [39]. These results are not in concordance with the results reported by Najar et al., who showed that geranyl acetate, an oxygenated monoterpene, was the major compound in *A. mexicana* [4,67].

#### 3.2.3. *Agastache foeniculum* Essential Oil

*A. foeniculum* is also reported to have high levels of estragole. The composition of *A. foeniculum* volatile oil recorded in the literature varies greatly, and these variations appear to be related to the provenance of the plant material. This suggests that *A. foeniculum* has several chemotypes.

Most studies that focused on the composition of *A. foeniculum* volatile oil showed that estragole was the volatile compound found in the highest concentration [56,57,58,59,60,68,69,70,71]. Besides estragole, other phenylpropanoid compounds (methyl isoeugenol [68], chavibetol [71], chavicol, eugenol [56]), as well as monoterpenes (1,8-cineole [58,70], limonene [56,57,59,71], pulegone, β-ocimene, bornyl acetate, geraniol, *trans*-carvone oxide [59]), sesquiterpenes (β-caryophyllene [68,71], spathulenol, caryophyllene oxide [59]), and non-terpene compounds (benzaldehyde, pentanol [56], 1-octen-3-ol [58]) have been identified.

As expected, the composition of the volatile oil depends on the part of the plant used. Thus, estragole was found in higher concentrations in the volatile oil of aerial parts (80–93%) and less in the essential oil extracted from leaves (18–30%) [56]. Also, estragole content was highest when the aerial parts were harvested at full bloom [60]. Irrigation regime [72], type of fertilizer used [73], and type of extraction procedure [74] were also found to influence the composition of *A. foeniculum* volatile oil. The volatile oil obtained using hydro distillation was approximately three times higher than that achieved from nitrogen purging. The distilled oils contained less benzaldehyde, octanone, limonene, and ocimene and higher boiling-point chemicals, particularly sesquiterpenes. The estragole concentrations of the oils and headspace were quite similar. Although this is an older study, it provides valuable information regarding the influence of the type of extraction on the composition of the essential oil [74]. Sourestani et al. showed that the relative quantities of the compounds in *A. foeniculum* volatile oil did not change as a result of drying procedures, storage, or distillation durations. The authors conclude that air drying (25 °C) and rapid extraction of essential oil using 2 h distillation are the optimal post-harvest treatments to optimize the amount of essential oil [57].

#### 3.2.4. *Agastache astromontana* Essential Oil

The major compounds in both the leaves and flowers of *Agastache astromontana* ‘Pink Pop’ volatile oil were linalool, bornyl acetate, and camphene. However, essential oils extracted from flowers were richer in bornyl acetate, whereas oils extracted from leaves were richer in linalool [2].

#### 3.2.5. *Agastache aurantiaca* Essential Oil

Studies have shown that *Agastache aurantiaca* volatile oil is mainly composed of oxygenated monoterpenes (88.8%), with pulegone as the major compound found in concentrations ranging from 76.7% to 77.9% [4,67].

#### 3.2.6. *Agastache* ‘Blue Boa’ Essential Oil

*Agastache* ‘Blue Boa’ volatile oil has a high concentration of oxygenated monoterpenes, with pulegone as the predominant compound (33.8%) [67]. The same results were reported by Najar et al., who confirmed that pulegone was the major compound in *A.* ‘Blue Boa’ (82.4%) [4]. On the contrary, Wilson et al. found that α-limonene (80.3%), β-caryophyllene (2.0%), and δ-cadinene (3.26%) were the major compounds in *A.* ‘Blue Boa’ leaves [75].

#### 3.2.7. *Agastache* ‘Arcado Pink’ Essential Oil

Hydrocarbonated sesquiterpenes (germacrene D and β-caryophyllene) were the main compounds in *A.* ‘Arcado Pink’ leaves (92.2%) [67]. Another study showed that the *A.* ‘Arcado Pink’ profile was characterized by a high concentration of both pulegone (monoterpene) and β-caryophyllene (sesquiterpene) in similar amounts (36.5% pulegone and 20.4% β-caryophyllene) [4].

#### 3.2.8. *Agastache scrophulariifolia* Essential Oil

There were few reports on the composition of *A. scrophulariifolia* volatile oil. Isomenthone (49.7%) and pulegone (19.8%) were reported as the major compounds in *A. scrophulariifolia* volatile oil from leaves [61,76].

Some references regarding the composition of essential oils are older [75,76], but given the context in which the evidence is limited, they bring important information regarding the chemical composition of some less investigated *Agastache* species, such as *Agastache* ‘Blue Boa’ and *Agastache scrophulariifolia.*

## 4. The Evaluation of Therapeutic Properties

Several studies focused on the evaluation of the biological properties of *Agastache* species. The essential oils isolated from *A. rugosa* and *A. foeniculum* demonstrated cytotoxic properties [26,50,51], while *A. rugosa* extracts showed anti-inflammatory effects [6,21], anti-adipogenic and anti-atherosclerotic properties [17,18], as well as anti-osteoporotic potential [19] and antioxidant properties [12].

*A. mexicana* extracts showed cardioprotective potential [1], antinociceptive [3], spasmolytic [38], and anxiolytic effects [28].

The essential oils isolated from several *Agastache* sp. had antibacterial [48,50], antifungal [66,68], and insecticidal activities [58,70].

### 4.1. Cytotoxic Activity

*A. rugosa* and *A. foeniculum* volatile oils were found to display cytotoxic potential against a panel of cancerous cell lines, and their activity is often explained by the presence of certain active compounds. In this regard, *A. rugosa* essential oil was cytotoxic against two hormonally dependent cancer cell lines, breast cancer (MCF-7) [77] and prostate cancer (LNCaP) [51]. As a particularity of the study, the essential oil obtained using steam distillation was further extracted with diethyl ether to concentrate the active compounds and thus obtain a cytotoxic effect at a lower dose [77]. *A. rugosa* volatile oil also reduced the viability of lung (A549) [77] and melanoma (B16) cell lines [51]. Patchouli alcohol, one of the active compounds of *A. rugosa* essential oil (45.70%), also displayed cytotoxicity against LNCaP and B16 cells [51].

*A. rugosa* volatile oil obtained from leaves and flowers had a time- and dose-dependent cytotoxic action on the gastric cancer line SGC-7901. Also, estragole and pulegone, the main components of the essential oil, expressed antiproliferative properties in the SGC-7901 cell line [78]. Another study also confirmed the cytotoxic effect of *A. rugosa* volatile oil against the gastric cancer cell line SGC-7901 [50].

Agastaquinone, a diterpenoid isolated from the roots of *A. rugosa,* and its oxime derivative demonstrated nonspecific cytotoxic activity against several human cancer cell lines, including non-small cell lung cancer (A549), ovarian cancer (SK OV-3), melanoma (SK-MEL-2), central nervous system cancer (XF498), and colon cancer (HCT15) [20]. Agastinol and agastenol, two lignans isolated from *A. rugosa*, inhibited apoptosis in U937 leukemia cells with IC_50_ values of 15.2 and 11.4 µg/mL, respectively [35].

The volatile oil extracted from *A. foeniculum* “Aromat de Buzău” was cytotoxic against the MCF-7 breast cancer cell line at concentrations greater than or equal to 0.2 µg/mL [26]. In another study, estragole, the main component of *A. foeniculum* volatile oil, showed dose-dependent cytotoxic activity on the MCF-7 breast cancer cell line (IC_50_ of 74 μg/mL), inducing apoptotic changes in the cancerous cell [79].

### 4.2. Anti-Inflammatory Activity

TRPA1 (transient receptor potential ankyrin1) and TRPV1 (transient receptor potential vanilloid 1) are receptors involved in processes such as pain and inflammation. TRPA1 and TRPV1 agonists can act as anti-inflammatory agents. *A. rugosa* stem and leaf extracts activated both receptors in vitro, indicating that *A. rugosa* could be associated with an anti-inflammatory effect. The authors suggested that the effect of the extract could be attributed to *trans*-*p*-methoxy cinnamaldehyde, L-carveol, methyl eugenol, *p*-anisaldehyde, and 4-allylanisole, which acted as TRPA1 agonists [6]. However, these volatile constituents are not usually found in extracts, but rather in the composition of essential oils. Thus, the effect of *A. rugosa* extract may be due to other active compounds.

In fact, several active compounds isolated from *A. rugosa* extract demonstrated anti-inflammatory activity, reducing prostaglandin E2 levels in lipopolysaccharide (LPS)-treated RAW 264.7 macrophages: (3*R*,7*R*)-tuberonic acid-12-*O*-[6′-*O*-(*E*)-feruloyl]-β-D-glucopyranoside, salicylic acid-2-*O*-[6′-*O*-(*E*)-feruloyl]-β-D-glucopyranoside, chavicol-1-*O*-(6′-*O*-methylmalonyl)-β-D-glucopyranoside, and other derivatives [21].

*A. rugosa* is a component of GHJGS (gwakhyangjeonggi-san), an herbal plant mixture used in traditional Korean medicine. It has anti-inflammatory and antioxidant effects in vitro in RAW 264.7 macrophages. In lipopolysaccharide (LPS)-stimulated macrophages, GHJGS significantly reduced the production of proinflammatory cytokines such as TNF-α (tumor necrosis factor-α), IL-6 (interleukin-6), and prostaglandin E2 [80].

Furthermore, *A. rugosa* essential oil exhibited in vivo anti-inflammatory activity in an adjuvant-induced arthritis rat model, reducing the level of pro-inflammatory markers such as TNF-α, IL-1, and IL-6 [51]. These findings suggest that *A. rugosa* contains a variety of bioactive chemicals with anti-inflammatory potential and could be beneficial for the treatment of pathologies associated with an inflammatory process.

### 4.3. Anti-Adipogenic and Anti-Atherosclerotic Activity

*A. rugosa* ethanolic extract and tilianin exhibited anti-lipogenic and anti-adipogenic effects on 3T3-L1 adipocytes. Tilianin caused a dosage-dependent decrease in lipid accumulation, whereas *A. rugosa* extract consistently decreased lipid accumulation regardless of dose, demonstrating that both tilianin and *A. rugosa* extract have anti-obesity effects in vitro. The researchers also found significant decreases in the levels of factors and proteins responsible for activating the process of adipogenesis, such as PPAR, C/EBP, SREBP1, and others [17]. Another study also confirmed that *A. rugosa* ethanolic extract decreased lipid accumulation by 20.4% in 3T3-L1 adipocytes [81].

Lee et al. showed that *A. rugosa* aqueous extract exerted an in vitro anti-atherosclerotic effect by inhibiting the proliferation of aortic vascular smooth muscle cells (VSMC). Also, by increasing the levels of the cyclin-dependent kinase inhibitors p21^WAF1/CIP1^ and p27^KIP1^, *A. rugosa* extract produced cell cycle arrest at the G0/G1 transition phase. Although both rosmarinic acid and caffeic acid were found in high concentrations in *A. rugosa* extract, only rosmarinic acid showed specific action on the inhibition of DNA synthesis and cell cycle progression during VSMC proliferation [18].

The in vitro anti-atherogenic effect of *A. rugosa* extract was also confirmed by in vivo studies. Thus, the administration of *A. rugosa* methanolic extract lowered plasma cholesterol levels in mice. The study found that *A. rugosa* extract therapy reduced the accumulation of macrophages in atherosclerotic lesions. To better understand the mechanism of action, the study also investigated the effects of tilianin on cultured human umbilical vein endothelial cells (HUVECs). The results showed that tilianin reduces TNF-K-induced VCAM1 expression by 74% in HUVECs, an early phase in atherosclerosis pathogenesis. Also, tilianin administration significantly reduced the activity of NF-kB, another important factor in the atherosclerosis process [82].

The study performed by Jun et al. concluded that *A. rugosa* volatile oil administration had a hypocholesterolemic effect in vivo, significantly decreasing LDL and triglyceride levels in mice. The essential oil was also effective in reducing the oxidation of LDL and up-regulating the expression of LDL receptors. Also, the administration of *A. rugosa* volatile oil down-regulated the expression of two transcription factors in the intrahepatic metabolism of cholesterol, SREBF-1 and SREBF-2, named sterol regulatory element binding factor [49].

### 4.4. Cardioprotective Activity

*A. mexicana* is traditionally used in herbal Mexican medicine for the treatment of hypertension [1,83]. Given the lack of scientific data that can support the empirical use of the plant, studies have investigated the cardioprotective effects of *A. mexicana* extracts and their main components using in vitro and in vivo models.

Tilianin isolated from *A. mexicana* exhibited vasorelaxant and antihypertensive effects when administered to male Wistar rats. Tilianin produced relaxation in concentration- and endothelium-dependent and independent manners in rat thoracic aortic rings. The mechanisms of action involved were NO/cGMP pathway modulation and the opening of K channels [1]. Furthermore, the same authors pointed out that extracts with a high concentration of tilianin have the best vasorelaxant effect, and the optimum method to obtain a high concentration of tilianin is by maceration with methanol, utilizing plant material dried at 50–100 °C [84]. Another study concluded that *A. mexicana* callus extract has vasorelaxant activity on the aortic ring. Interestingly, the callus extract with the lowest tilianin level had the best vasorelaxant effect when compared with the wild plant. This could be due to the presence of various secondary metabolites that are most likely only formed in in vitro cultures [85]. Regarding the safety profile, tilianin in doses less than 1000 mg/kg did not pose any visible toxicological effects when administered to mice: ED_50_ (53.51 mg/kg) for antihypertensive effect and LD_50_ (6624 mg/kg) were determined [86].

Some authors suggested that ursolic acid could also be responsible for the antihypertensive activity of *A. mexicana*. The dichloromethane extract produced a considerable vasorelaxant effect ex vivo in a rat aorta ring model. Moreover, the isolated ursolic acid showed antihypertensive action in hypertensive rats. Thus, the available studies support the empirical use of *A. mexicana* as an antihypertensive drug in Mexican traditional medicine [83].

Noteworthy, Cao et al. (2017) showed that *A. rugosa* extract also displayed procoagulant activity, while the isolated main components of *A. rugosa* extract, acacetin and tilianin, had significant anticoagulant activity in vitro [23]. Further investigation of the in vivo effects could lead to a better understanding of the possible use of *Agastache* sp. extracts in the management of bleeding disorders.

### 4.5. Antidiabetic Activity

Acacetin (5,7-dihydroxy-4′-methoxyflavone), one of the main constituents of *A. rugosa* extract, showed antidiabetic potential in vitro. Acacetin increased glucose absorption in cultured muscle cells via an insulin-independent pathway, GLUT4 translocation. Moreover, acacetin-induced GLUT4 translocation was mediated via intracellular reactive oxygen species (ROS) generation [87].

Another study aimed to determine the antidiabetic effect of tilianin (Acacetin 7-glucoside) in diabetic rats. Tilianin was isolated from the methanolic extract of *A. mexicana*. Tilianin administration (60 mg/kg body weight) for 10 days induced a significant antihyperglycemic effect at day 6. The administration of tilianin decreased IL-1b and IL-18 expression in adipose tissue, two pro-inflammatory mediators that can affect insulin sensitivity [88].

Thus, *Agastache* sp. extracts could be investigated in terms of antidiabetic potential, corroborating the presence of tilianin and acacetin, two active compounds with antidiabetic potential.

### 4.6. Hepatoprotective Activity

Cho et al. found that acacetin, isolated from aerial parts of *A. rugosa*, has a hepatoprotective effect in vivo against fulminant hepatic failure caused by D-galactosamine and lipopolysaccharide in mice. Acacetin pretreatment significantly increased the survival rate, reducing the level of seric aminotransferase, a biomarker for hepatocellular injury. Acacetin pretreatment also reduced the level of the pro-inflammatory tumor necrosis factor-α (TNF-α) and increased the level of IL-6, a pleiotropic cytokine involved in hepatic homeostasis. The study concluded that acacetin inhibits D-galactosamine/lipopolysaccharide-induced hepatocellular damage, which could be relevant in future therapeutic applications for treating liver illnesses [89]. It is important to consider that following oral administration of *A. rugosa* extract in rats, acacetin was found in high concentrations in urine and feces, thus representing the main active metabolite [90]. Apart from acacetin, the unmetabolized main components of *A. rugosa*, tilianin, and rosmarinic acid were found in very low concentrations in urine and feces, indicating that these compounds are extensively converted to acacetin in rats. The hydrolysis of acacetin-*O*-glucoside (tilianin) to acacetin appears to play a significant role in the metabolism of acacetin-derivative secondary metabolites in *A. rugosa* [90]. Corroborating with the hepatoprotective effect of acacetin [89], *Agastache* sp. extracts warrant further investigation of the hepatoprotective potential in vivo.

Using the same animal model, Cho et al. demonstrated that β-caryophyllene, a sesquiterpene compound from *A. rugosa* essential oil, has hepatoprotective potential in acute hepatic failure. The intraperitoneal administration of β-caryophyllene prior to the induction of hepatic failure led to a reduction in the level of serum aminotransferase, tumor necrosis factor-α, and interleukin 6 [91].

Thymoquinone is an active aromatic compound found in *Agastache* sp. One study found that thymoquinone has a hepatoprotective effect on acetaminophen-induced acute liver injury. Administration of thymoquinone in rats reduced the levels of ALT and AST and also restored the levels of GSH, which has a significant role in the detoxification of acetaminophen. The authors also concluded that the hepatoprotective effect of thymoquinone is mediated through suppression of the MAPK signaling pathway [92].

Taken together, these studies promote the future investigation of the hepatoprotective potential of *Agastache* sp. extracts and volatile oils against acute liver injury.

### 4.7. Anti-Osteoporotic Activity

*A. rugosa* extract showed anti-osteoporotic potential both in vitro and in vivo. *A. rugosa* ethanolic extract stimulated the differentiation of MC3T3-E1 pre-osteoblasts [19]. Also, *A. rugosa* extract reduced the expression of NO-synthetase and inhibited NO production in ROS 17/2.8 osteoblasts. Excess NO production has been associated with osteoblast apoptosis and bone loss. Thus, *A. rugosa* leaf extract prevents NO-mediated apoptosis in vitro and may be beneficial in the prevention of osteoporosis and other inflammatory bone conditions [93].

In vivo, *A. rugosa* ethanolic extract reversed postmenopausal osteoporosis induced in an animal model by ovariectomy [19]. It has been shown that the anti-osteoporotic effect is also mediated by changes in the gut microbiome, by increasing the number of *Deferribacteres*, *Proteobacteria*, and *Bacteroidetes* and decreasing the number of *Firmicutes* bacteria [19]. Similar results were obtained by Jang et al., who discovered that *A. rugosa* aqueous extract reduced ovariectomy-induced bone loss in mice by suppressing RANK ligand—a marker of the osteoclast differentiation. The extract inhibited the expression of cellular oncogene fos (c-Fos) and nuclear factor NFATc1, key factors in osteoclastogenic transcription. The results support the possibility of using the aqueous extract of *A. rugosa* in the prevention and treatment of post-menopausal osteoporosis by suppressing osteoclastogenesis [94].

### 4.8. Anti-Nociceptive Activity

*A. mexicana* has been traditionally used in Mexican folk medicine for the treatment of pain. Decoction of *A. mexicana* aerial parts is empirically indicated for various pain disorders such as stomachache, colic, and intestinal pain, as well as to enhance digestion [3]. However, little scientific evidence supporting its use has been reported. Nevertheless, the latest research points towards the efficacy of *A. mexicana* extracts and volatile oils in the treatment of nociception.

*A. mexicana* ssp. *mexicana* and ssp. *xolocotziana* volatile oils showed anti-nociceptive activity in vivo, reducing abdominal pain in an animal model. The effect of the volatile oils from both subspecies could be explained by the presence of limonene and pulegone. Limonene demonstrated the strongest anti-nociceptive action in the study. It also significantly reduced intestinal inflammatory cytokines in a drug-induced ulcerative colitis model, providing gastroprotection against gastric injury. The results of the study support the traditional use of *A. mexicana* in the treatment of gastrointestinal conditions, showing the therapeutic potential of monoterpenes in the treatment of ulcers, colitis, and abdominal pain [95]. Another study confirmed the antinociceptive effect of pulegone in vivo. The tests that were used included the hot plate test and the formalin test. As a result, the authors suggested that pulegone could contribute to the antinociceptive effect of *Agastache* sp. essential oils [96].

*A. mexicana* extracts in hexane, ethyl acetate, and methanol inhibited the abdominal constriction generated by 1% acetic acid injection. Hexane and ethyl acetate extracts showed the highest antinociceptive efficacy. Although methanol extract was obtained in major yield, its antinociceptive effect was inferior to that of hexane and ethyl acetate extracts, suggesting less participation of the polar compounds in this type of nociception [97].

One study indicated that the antinociceptive effect of *A. mexicana* extracts could be partially explained by the presence of ursolic acid, a triterpene found in concentrations of 130.7 mg/g (ethyl acetate extract) and 20.3 mg/g (methanolic extract). Ursolic acid exhibited significant antinociceptive potential in different animal models, including the formalin test and the writhing test. Its efficacy was demonstrated in both neurogenic and inflammatory nociception, while its mechanism of action could involve the presence of cGMP and TRPV1 receptors [3]. These results were confirmed by another study, which showed that different types of *A. mexicana* extracts (hexane, ethyl acetate, and methanol), along with acacetin and ursolic acid, showed anti-nociceptive activity using the writhing test in mice [98].

Thus, the traditional use of *A. mexicana* as a pain reliever is sustained by in vivo studies that confirmed its antinociceptive effect in different types of nociception models.

### 4.9. Spasmolytic Activity

*A. mexicana* ssp. *xolocotziana* extract displayed spasmolytic properties on guinea pig ileum pre-contracted with KCl, while *A. mexicana* ssp. *mexicana* had a spasmogenic effect. Both subspecies are used in folk medicine for abdominal pain and other gastrointestinal diseases, but when administered in vivo, only ssp. *xolocotziana* has been shown to exhibit a spasmolytic effect. The mechanism of action could involve nicotinic receptor agonism and blockade of calcium channels. Qualitative and quantitative variances in the composition of each subspecies could be accountable for their distinct pharmacological effects [38].

Using the same animal model and inducing ileum contractions with KCl, another study found that acacetin and ursolic acid, two active components from *A. mexicana* extract, may be responsible for the spasmolytic effect of *A. mexicana* [98].

### 4.10. Gastroprotective Activity

*A. rugosa* 70% ethanolic extract (100 mg/kg/day) showed gastroprotective effects in an acute gastritis mouse model induced by administration of HCl/EtOH (clorhidric acid/ethanol). The gastroprotective effect was explained by the increased secretion of gastric mucus and the inhibition of the infiltration of inflammatory cells. At the molecular level, *A. rugosa* affected some signaling pathways involved in the cellular inflammatory response (PI3K/AKT and NF-kB). These results confirm the use of Korean mint as a medicinal plant in the treatment of digestive disorders such as dyspepsia, nausea, and vomiting [14].

### 4.11. Anxiolytic Activity

*A. mexicana* is traditionally used as a natural remedy for the treatment of anxiety disorders [99]. Thus, researchers evaluated the in vivo anxiolytic effect of *A. mexicana* extract and tilianin, one of the major bioactive compounds of the extract. The results showed that methanolic crude extract (10 to 300 mg/kg, i.p. and 300 mg/kg p.o.) and tilianin (30 to 300 mg/kg, i.p. and 300 mg/kg, p.o.) had anxiolytic effects when administered to mice. The mechanism of action could involve GABA_A_ receptors, considering that the anxiolytic effect did not occur in the presence of flumazenil. These results demonstrated that tilianin is one of the main components of the methanolic extract responsible for the anxiolytic activity of *A. mexicana* and requires further research involving clinical studies [100].

Other researchers found that aqueous extracts of *A. mexicana* ssp. *mexicana* and *xolocotziana* exhibited anxiolytic-like activities at low doses (0.1 to 10.0 mg/kg), but at larger doses (above 100 mg/kg), they showed sedative activity that impacted general activity and motor coordination. The aqueous extract of ssp. *mexicana* appears to be more potent than that of ssp. *xolocotziana*, as it causes anxiolytic-like effects and sedative effects at lower doses [28]. Interestingly, an older study found that the effects of *A. mexicana* water extract are more consistent with an anxiogenic-like property than an anxiolytic-like property [101], but these results were not confirmed by other studies. These findings support the use of *A. mexicana* ssp. *mexicana* and ssp. *xolocotziana* in traditional medicine as anxiolytic, sedative, and tranquilizing agents.

Furthermore, a study showed that exposure to *A. rugosa* essential oil produced significant changes in EEG activity, stimulating brain wave activity and increasing spectral edge frequency by 50% of alpha spectra values. Also, exposure to volatile oil reduced the absolute and relative theta power spectra. These EEG changes suggest that *A. rugosa* essential oil can be used in aromatherapy for positive psychophysiological conditions [45].

### 4.12. Antioxidant Activity

Several studies evaluated the in vitro antioxidant effects of *Agastache* sp. extracts and volatile oils, and they demonstrated good antioxidant activity against different free radicals.

Studies showed that *A. rugosa* extract had DPPH (2,2-diphenyl-1-picrylhydrazyl) and ABTS (2,2′-azino-bis(3-ethylbenzothiazoline-6-sulfonic acid)) radical scavenging activity [12,81]. The essential oil from *A. rugosa* also displayed radical scavenging potential against DPPH, with an antioxidant activity of 8.79 µg/mL [51]. Regarding the mechanism of action, researchers found that *A. rugosa* leaf extract manifested its antioxidant activity by increasing the expression of the HO-1 (heme oxygenase 1) protein, an enzyme that protects against oxidative stress. The study showed that *A. rugosa* extract protects RAW264.7 macrophages against H_2_O_2_-induced oxidative stress and could be useful for the treatment of pathologies associated with cellular oxidative stress [102]. Furthermore, Gwakhyangjeonggi-san, a traditional herbal mixture that also includes *A. rugosa*, increased HO-1 expression and prevented the formation of reactive oxygen species in RAW 264.7 cells [80].

*A. rugosa* honey presents ferric-reducing antioxidant power of 3.61 ± 0.02 µmol TE/g (TE = Trolox equivalents) [103] and DPPH radical scavenging activity (IC_50_ = 0.77 ± 0.02 mg/mL), with a significant positive correlation between the total antioxidant capacity and the total phenol content of the tested bee product [52].

*A. foeniculum* essential oil and extracts exhibited good antioxidant activity. The essential oil was effective against DPPH radicals, inhibiting 77.88% of DPPH radicals at a concentration of 10 μL/mL [68]. *A. foeniculum* extract showed DPPH radical scavenging activity, cupric-reducing antioxidant capacity, and ABTS radical scavenging activity [25,27,37]. The antioxidant activity measured by the ABTS assay was 67.3 ± 1.9 µmol TE/g (TE = Trolox equivalents) [37].

The DPPH scavenging activity of *A. aurantica* flowers extract is comparable to previously published values from different *Agastache* flowers [104].

Plant phytochemical composition influences antioxidant activity. Typically, phenolic chemicals contribute significantly to antioxidant activity. The antioxidant activity, as evaluated by the DPPH assay, was shown to be highly correlated with the level of hydroxycinnamic acids (r = 0.77, *p* ≤ 0.05) as well as the total concentration of phenolic compounds (r = 0.74, *p* ≤ 0.05). There was also a strong association between antioxidant activity (DPPH assay) and flavonoid concentration (r = 0.58, *p* ≤ 0.05). A stronger link was also shown between antioxidant activity, as determined by the ABTS assay, and the total amounts of hydroxycinnamic acids and phenolic compounds [37].

Taken together, these studies show that *Agastache* sp. extracts and volatile oils have good antioxidant activity in different in vitro assays.

### 4.13. Enzyme Inhibition Activity

The inhibitory capacity of *Agastache* sp. extracts on the activity of some enzymes has potential applications in many fields.

*A. rugosa*’s ethanolic extract inhibited xanthine oxidase (XO). Acacetin, one of the major compounds isolated from the extract, also exhibited xanthine oxidase inhibitory action (IC_50_ = 0.58 M), which is lower compared with allopurinol, routinely used as a xanthine oxidase inhibitor. Thus, *A. rugosa* extract could act as a functional component and natural medicine for the treatment of hyperuricemia and gout [32].

Lee et al. showed that acacetin and its derivative, acacetin 7-*O*(6-*O*-malonylglucoside), two active compounds isolated from the methanolic extract of *A. rugosa* leaves, had inhibitory activity on MAO-A and MAO-B, two enzymes implicated in the degradation of amines, including dopamine. Thus, acacetin and its derivative could be further tested for their potential as MAO inhibitors, with application in the treatment of some neurological disorders, including Parkinson’s disease [32].

*A. foeniculum* extract inhibited α-amylase and α-glucosidase, enzymes related to carbohydrate digestion. Amylase inhibitory activity was highly correlated with hydroxycinnamic acid concentration and moderately correlated with flavonoid concentration [37]. The inhibition of α-amylase and α-glucosidase could be beneficial in the treatment of type 2 diabetes; thus, further investigation of the antidiabetic potential of *A. foeniculum* extract is needed.

### 4.14. Antibacterial Activity

The essential oil obtained from *A. rugosa* flowers and leaves was found to have moderate to high antimicrobial activity. A strong in vitro inhibitory effect against *Staphylococcus aureus* (*S. aureus*—MIC 21 µg/mL) and *Escherichia coli* (*E. coli*—MIC 21 µg/mL) was noticed in the case of the essential oil from flowers, while the essential oil from leaves strongly inhibited *E. coli* (MIC 9.4 µg/mL). Both products were able to inhibit biofilm formation [50]. The antibacterial activity of the essential oil against *S. aureus* and *E. coli* was also confirmed by another study, which in addition reported antimicrobial activity against two other Gram-negative bacteria (*Salmonella enteritidis* and *Pseudomonas aeruginosa).* The antibacterial capacity could be attributed to estragole, the major compound of essential oil, but also to other trace volatile compounds such as terpenoids, including dl-limonene, linalool, β-caryophyllene, and germacrene-D [48]. *E. coli* was also susceptible to the action of *A. rugosa* methanolic extract, together with *Aeromonas hydrophila*, *Aeromonas salmonicida*, *Staphylococcus haemolyticus,* and *Cronobacter sakazakii* [15].

Antimicrobial activity evaluation showed that *A. foeniculum* essential oil had a moderate effect on some Gram-positive (*Curtobacterium flaccumfaciens*, *Listeria monocytogenes*, *Bacillus subtilis*) and Gram-negative bacteria (*Salmonella* sp., *Proteus vulgaris*, *Klebsiella pneumoniae*), with MICs ranging from 0.157 to 2.50 µL/mL. [68]. Regarding the antibacterial capacity of the extracts, silver nanoparticles obtained from *A. foeniculum* aqueous extract showed in vitro properties against several bacterial strains (*Staphylococcus aureus*, *Staphylococcus haemolyticus*, *Klebsiella pneumoniae,* and *Streptococcus pneumoniae*) [36].

### 4.15. Antifungal Activity

Shin et al. found that *A. rugosa* essential oil fraction and estragole exhibited antifungal activity against *Aspergillus* and *Candida* species (MICs ranging from 2.5 and 10 mg/mL). Also, essential oil and estragole displayed synergism with ketoconazole against *Blastoschizomyces capitatus*, a rare fungus that causes serious infections in immunocompromised patients [105]. These results were also confirmed by other studies, which pointed out the synergism of action between the estragole isolated from *A. rugosa* and ketoconazole against *Candida* species (*C. albicans*, *C. utilis*). However, this synergism was not confirmed for the combination of estragole and amphotericin B [106]. *C. albicans* was also susceptible in vitro to the action of *A. foeniculum* essential oil [68].

Another study evaluated the antifungal capacity of essential oil from *A. rugosa* against *Trichophyton* species. Synergistic effects were noticed between *A. rugosa* essential oil and estragole with ketoconazole against the studied *Trichophyton* species, with fractional inhibitory concentration indices that ranged from 0.05 to 0.27 [107].

Juárez et al. showed that *A. mexicana* ssp. *xolocotziana* volatile oil had antifungal activity against a panel of fungal strains, including *Aspergillus* sp., *Penicillium* sp., and *Eupenicillum* sp., with MICs ranging from 0.3 to 30 g/mL [66].

Other researchers investigated the antifungal properties of *Agastache* honey in comparison to other commercial honeys. *Agastache* honey showed superior antifungal activity against dermatophytes (*T. mentagrophytes* and *T. rubrum*) and *C. albicans*. *Agastache* honey contained several volatile compounds with antifungal activity, whereas other honeys contained few antifungal compounds. Phenol-2,4-bis(1,1-dimethylethyl), estragole, nonanoic acid, and ethyl ester were found as chemical markers in *Agastache* honey. *Agastache* honey products could be developed for topical use against fungal skin infections [44].

### 4.16. Insecticidal Activity

*A. foeniculum* volatile oil displayed strong insecticidal activity against *Tribolium castaneum* and *Rhyzopertha dominica* [70]. Moreover, the essential oil of *A. foeniculum* was found to be effective as a natural pesticide against *Oryzaephilus surinamensis* and *Lasioderma serricorne* [58]. These findings suggest that *A. foeniculum* volatile oil can be used to manage and reduce the negative impacts of modern insecticides.

### 4.17. Anti-Photoaging Activity

Some in vitro studies investigated the protective potential of *A. rugosa* extract against photoaging induced by UV-B radiation in HaCaT keratinocytes. The results of the studies showed that *A. rugosa* extract inhibited the activity of elastase and hyaluronidase, enzymes associated with the skin aging process [29]. Also, the extract presented an effective radical scavenging activity against DPPH, superoxide, nitrite [29], and ABTS^+^ [30] free radicals. Moreover, *A. rugosa* extract reduced the level of NO, an important intracellular pro-inflammatory mediator [29,108], and the level of reactive oxygen species [29,30,108,109], thus demonstrating anti-inflammatory and antioxidant potential.

*A. rugosa* extract’s anti-UV-B protective properties are augmented when using probiotic fermentation [108,109]. Both non-fermented and fermented extracts significantly reduced the level of ROS induced by UVB in keratinocytes and enhanced the activity of glutathione and superoxide dismutase, two antioxidant components involved in protection against oxidative stress [30,109].

Later, other researchers investigated the anti-photoaging effect of *A. rugosa* extract in an animal model. The extract was given orally to hairless mice at doses of 100 or 250 mg/kg/day for 12 weeks, along with UVB exposure. UVB-induced wrinkle formation, epidermal thickening, erythema, and hyperpigmentation have been histologically improved. The extract also restored skin moisture by improving skin hydration and transepidermal water loss, increasing hyaluronic acid levels, and significantly increasing collagen density [110]. Thus, these findings suggest that *A. rugosa* extract could be used as a preventive and therapeutic agent against photoaging and support the extract’s cosmetic value.

### 4.18. Effects on the Respiratory System

A herbal mixture containing *Glycyrrhiza glabra*, *Agastache rugosa,* and tilianin significantly reduced neutrophil levels in the lungs and bronchoalveolar lavage fluid in an animal model of chronic obstructive pulmonary disease, thus showing potential in the management of this severe pathology [111].

*A. mexicana* ssp. *mexicana* volatile oil produced relaxation of the trachea smooth muscle, attenuating carbachol- and histamine-induced contractions. The study also suggested that the mechanism of action involved calcium channel blockade and that estragole and D-limonene could be responsible for the relaxant effect of the essential oil [65]. Also, *A. mexicana* extract had a relaxant effect on rat-isolated trachea contracted with carbachol [112]. Thus, *A. mexicana* extract and volatile oil could be further investigated in terms of their anti-asthmatic potential.

## 5. Conclusions and Future Perspectives

In this paper, we have performed a comprehensive review of the literature on the phytochemical profile and pharmacological properties of several species from the *Agastache* genus, a small genus of aromatic plants from the Lamiaceae family. Studies on *Agastache* sp. have seen significant progress in the last decade. Studies evaluating the chemical composition and therapeutic potential pointed out that *Agastache* sp. represent valuable natural sources of bioactive compounds. *Agastache* sp. extracts contain mainly flavonoids (tilianin, acacetin, apigenin, quercetin) and phenolic acids (rosmarinic acid, chlorogenic acid, caffeic acid). Tilianin (acacetin-7-*O*-glucoside) and its aglycone, acacetin, were identified as major compounds in the hydroalcoholic extracts of *Agastache* sp. Many of the therapeutic effects of the extracts have been attributed to the presence of these secondary metabolites. Some of the most important pharmacological effects of the extracts include anti-adipogenic, anti-atherosclerotic, cardioprotective, anti-diabetic, anti-osteoporotic, and hepatoprotective properties. The anti-atherosclerotic and cardioprotective effects of the extracts have been explained by the presence of tilianin, a glycosidic flavonoid with therapeutic potential in the cardiovascular field. Tilianin exhibited anti-lipogenic, anti-atherosclerotic, antihypertensive, and anticoagulant activity. Although these are promising results, most of the studies used in vitro models. To better understand the therapeutic potential of *Agastache* sp. extracts and their main components, in vivo studies are needed. Subsequently, standardization of the extracts of tilianin and providing a specific concentration of the active compound could enhance therapeutic efficacy and guarantee the quality of the extracts. Another option would be the structural modeling of tilianin with the development of structural analogues and the investigation of structure-activity relationships.

The volatile oils obtained from *Agastache* sp. contain mainly phenylpropanoids (estragole and methyl eugenol) and terpenoids (limonene, germacrene, and pulegone). Estragole is the major volatile compound reported in most of the studies; however, there are many chemotypes within the same species. Some chemotypes are characterized by the dominance of phenylpropanoids, while others are characterized by the dominance of terpenes. Thus, depending on the chemical composition, *Agastache* sp. volatile oils have demonstrated different pharmacological effects: cytotoxic, antimicrobial, anti-nociceptive, anti-inflammatory, and anti-atherosclerotic. Estragole also exhibited cytotoxic, antibacterial, and antifungal effects. Future research directions include testing the cytotoxic effect of volatile oils and estragole on other cancerous cell lines (colon, triple-negative breast, lung, and others) and investigating the cytotoxic potential of other *Agastache* species. The cytotoxic effect has been determined so far only for *A. rugosa* and *A. foeniculum*.

In the few studies conducted on animals, the authors do not report the adverse effects or toxicity of *Agastache* sp. volatile oils. Also, the dosage and mode of administration of the volatile oils are not always clearly mentioned. The extraction of the active volatile compounds from the essential oils and the investigation of their pharmacological effects may considerably enhance the therapeutic potential of the volatile oils.

Although research on *Agastache* species has seen a lot of progress in recent years, there are still many scientific gaps that require investigation. Of the 22 species of the genus, only a few of them have been characterized phytochemically and pharmacologically. Despite the promising therapeutic potential of some *Agastache* sp. extracts, volatile oils, or their active components, clinical studies are lacking. Moreover, some pharmacological properties have been tested only in vitro, and most of them were attributed to crude extracts and not to active compounds. Thus, the investigation of the pharmacological effects using animal models, the testing of the active compounds, and subsequently the standardization of extracts from these compounds represent important research directions.

In conclusion, *Agastache* species are aromatic plants considered natural sources of important bioactive compounds, representing potentially effective clinical drug candidates. Compared with other genera, *Agastache* genus is less investigated; thus, our present work gives a scientific foundation for future research and medicinal applications on the plants of this genus.

## Figures and Tables

**Table 1:** Identified polyphenolic compounds in *Agastache* sp. extracts.

Species/ Origin	Plant Part	Type of Extract	Major Compounds	Ref.
*A. rugosa*/ Poland	Leaves	Methanolic	Rosmarinic acid (25.81 mg/g d.w.), apigenin *O*-glucoside (15.61 mg/g d.w.), chlorogenic acid (7.24 mg/g d.w.), acacetin acylglycosyl (3.51 mg/g d.w.)	[9]
*A. rugosa*/Poland	Leaves	Methanolic	Rosmarinic acid (10.76 mg/g d.w.), tilianin (2.92 mg/g d.w.), chlorogenic acid (2.46 mg/g d.w.)	[10]
*A. rugosa*/Poland	Inflorescences	Methanolic	Rosmarinic acid (4.98 mg/g d.w.), tilianin (1.60 mg/g d.w.), chlorogenic acid (1.19 mg/g d.w.)	[10]
*A. rugosa*/Republic of Korea	Not mentioned	Ethanolic 70%	Rosmarinic acid, tilianin, acacetin, acacetin-7-*O*-(6″-*O*-malonyl)-β-D-glucopyranoside, isoagastachoside, acacetin-7-*O*-(2″-*O*-acetyl-6″-*O*-malonyl)-β-D-glucopyranoside	[11]
*A. rugosa*/Republic of Korea	Flowers, leaves, stem and roots	Methanolic 70%	Rosmarinic acid (407.89–742.49 mg/kg fresh sample), apigenin (194.77–1295.09 mg/kg fresh sample), calycosin (385.19–2061.83 mg/kg fresh sample), phloretin-hexoside (48.66–459.24 mg/kg fresh sample)	[12]
*A. rugosa*/Republic of Korea	Leaves and stem	Ethanolic 80%	Acacetin 7-*O*-(6″-*O*-malonyl)-β-D-glucopyranoside (10.06–14.30 mg/g d.w.), acacetin (0.8–1.5 mg/g d.w.), acacetin 7-*O*-(2″-*O*-acetyl)-β-D-glucopyranoside (0.11–1.56 mg/g d.w.),tilianin (0.16–0.43 mg/g d.w.), acacetin 7-*O*-(2″-*O*-acetyl-6″-*O*-malonyl)-β-D-glucopyranoside (0.05–0.35 mg/g d.w.), rosmarinic acid (0.031–0.29 mg/g d.w.)	[13]
*A. rugosa*/Republic of Korea	Flowers and seeds	Ethanolic 80%	Acacetin 7-O-(6″-*O*-malonyl)-β-D-glucopyranoside (6.44–34.25 mg/g d.w.), tilianin (0.56–4.1 mg/g d.w.), acacetin (0.44–3.37 mg/g d.w.), acacetin 7-*O*-(2″-*O*-acetyl)-β-D-glucopyranoside (0.04–4.4 mg/g d.w.), acacetin 7-*O*-(2″-*O*-acetyl-6″-*O*-malonyl)-β-D-glucopyranoside (0.06–1.19 mg/g d.w.), rosmarinic acid (0.07–0.47 mg/g d.w.)	[13]
*A. rugosa*/Republic of Korea	Leaves	Methanolic	Apigenin, acacetin, tilianin, acacetin 7-*O*-β-(3″-acetylglucopyranoside), acacetin 7-*O*-β-(6″-(E)-crotonylglucopyranoside)	[14]
*A. rugosa*/Republic of Korea	Flowers, leaves and stem	Methanolic	Caffeic acid (0.267–0.327 mg/g d.w.), chlorogenic acid (0.011–0.103 mg/g d.w.), catechin (0.004–0.148 mg/g d.w.), rutin (0.053–0.081 mg/g d.w.)	[15]
*A. rugosa*/Republic of Korea	Flowers	Methanolic	Tilianin (7.13–12.52 mg/g d.w.), rosmarinic acid (2.17–5.24 mg/g d.w.), quercetin (1.51–4.53 mg/g d.w.), acacetin (0.79–3.02 mg/g d.w.), chlorogenic acid (1.16–1.78 mg/g d.w.)	[16]
*A. rugosa*/Republic of Korea	Leaves	Ethanolic	Tilianin (21.14 ± 0.15 mg/g), acacetin (9.94 ± 0.08 mg/g), acacetin 7-*O*-(6″-*O*-malonyl)-β-D-glucopyranoside	[17]
*A. rugosa*/Republic of Korea	Not mentioned	Aqueous	Rosmarinic acid (100.89 mg/g), caffeic acid (30.04 mg/g)	[18]
*A. rugosa*/Republic of Korea	Aerial parts	Ethanolic 80%	Tilianin (38.29 mg/dry g), rosmarinic acid (25.81 mg/dry g), acacetin 7-*O*-(6″-*O*-malonyl)-β-D-glucopyranoside (11.90 mg/dry g), acacetin 7-*O*-(2″-*O*-acetyl)-β-D-glucopyranoside (10.32 mg/dry g), acacetin (7.88 mg/dry g), acacetin 7-*O*-(2″-*O*-acetyl-6″-malonyl)-β-D-glucopyranoside (3.40 mg/dry g)	[19]
*A. rugosa*/Republic of Korea	Roots	n-Hexane	Agastaquinone	[20]
*A. rugosa*/Republic of Korea	Aerial parts	Ethanolic 70%	Chavicol-1-*O*-(6′-*O*-acetyl)-β-D-glucopyranoside, chavicol-1-*O*-(6′-*O*-methylmalonyl)-β-D-glucopyranoside, salicylic acid-2-*O*-[6′-*O*-(*E*)-feruloyl]-β-D-glucopyranoside, (3R,7R)-tuberonic acid-12-*O*-[6′-*O*-(*E*)-feruloyl]-β-D-glucopyranoside, acacetin-7-*O*-(3″-*O*-acetyl-6″-*O*-malonyl)-β-D-glucopyranoside	[21]
*A. rugosa*/Republic of Korea	Flowers	Methanolic 80%	Tilianin (2.04–3.11 mg/g d.w.), rosmarinic acid (1.66–2.95 mg/g d.w.), acacetin (0.23–0.66 mg/g d.w.)	[22]
*A. rugosa*/ China	Aerial parts	Ethanolic 70%	Tilianin, acacetin, apigenin, methyl-hexadecanoate, ursolic acid, protocatechuic acid, β-sitosterol	[23]
*A. rugosa*/ China	Aerial parts	1-Butyl-3-methylimidazoliumbromide-methanol	Tilianin, acacetin	[24]
*A. foeniculum*/ Romania	Not mentioned	Ethanolic 80%	Isoquercitrin (283.22 µg/g d.w.), resveratrol (128.59 µg/g d.w.), quercetin (117.69 µg/g d.w), acacetin (52.83 µg/g d.w.), chlorogenic acid (49.88 µg/g d.w.), vanilin (40.96 µg/g d.w.)	[25]
*A. foeniculum*/ Romania	Aerial parts	Ethanolic	Genistein (2229.99 µg/g d.w.), quercetin (704.14 µg/g d.w.), hyperoside (58.89 µg/g d.w.), rutin (56.70 µg/g d.w.)	[26]
*A. foeniculum*/ Romania	Aerial parts	Ethanolic 70%	Apigenin-7-*O*-glycoside (16.15–18.50 mg/100 g dry plant), rosmarinic acid (6.45–8.12 mg/100 g dry plant), quercetin-3-*O*-glycoside (6.20–8.30 mg/100 g dry plant), quercetin (4.05–10.21 mg/100 g dry plant), luteolin-7-glycoside (2.10–2.22 mg/100 g dry plant)	[27]
*A. mexicana* ssp. mexicana/Mexico	Aerial parts	Aqueous	Acacetin 7-*O*-β-D-(6″-*O*-malonyl)-glucoside (31.47%), acacetin 7-*O*-β-glucoside (24.49%, luteolin 7-*O*-β-D-(6″-*O*-malonyl)-glucoside (15.22%), diosmetin 7-*O*-β-D-(6″-*O*-malonyl)-glucoside (13.95%), acacetin-7-*O*-β-glucoside-D-(2″-acethyl-6″malonyl) (12%)	[28]
*A. mexicana* ssp. xolocotziana/Mexico	Aerial parts	Aqueous	Diosmetin 7-*O*-β-D-(6″-*O*-malonyl)-glucoside (32.9%), acacetin 7-*O*-β-glucoside (22.42%), acacetin-7-*O*-β-glucoside-D-(2″-acethyl-6″malonyl) (11.21%), diosmetin 7-β-*O*-glucoside (5.14%), acacetin 7-*O*-β-D-(6″-*O*-malonyl)-glucoside (2.21%)	[28]

**Table 2:** Major volatile compounds in *Agastache* sp. essential oils.

Species/ Origin	Plant Part	Major Volatile Compounds	Ref.
*A. rugosa*/ Australia	Flowers	Estragole (96.74%), bisabolol, caryophyllene, germacrene D, limonene, methyl eugenol, muurolene, octen-3-ol	[43]
*A. rugosa*/ Australia	Flowers + Nectar	Estragole (97.16%), bicyclogermacrene, bisabolene, bisabolol, caryophyllene, germacrene D, gurjunene, humulene	[43]
*A. rugosa*/ Australia	Leaves	Estragole (94.35%), bicyclogermacrene, caryophyllene, germacrene D, humulene, limonene, methyl eugenol, murolene, octen-3-ol, octanone, octen-3-yl acetate	[43]
*A. rugosa*/ Australia	Mono-floral honey	Phenol, 2,4-bis(1,1-dimethylethyl) (12.77%), estragole (12.31%), nonanoic acid, ethyl ester (7.22%)	[44]
*A. rugosa*/ Republic of Korea	Aerial parts	Estragole (89.49%), D-limonene (3.40%), menthone (1.80%), pulegone (1.86%)	[45]
*A. rugosa*/ Republic of Korea	Aerial parts	Estragole, D-limonene, isopulegone, menthone, pulegone, β-caryophyllene, β-cubebene	[46]
*A. rugosa*/ Republic of Korea	Flowers and Leaves	Estragole (84.25%—leaves, 57.94%—flowers), *trans*-caryophyllene (1.00%—leaves; 2.05%—flowers), L-limonene (0.51%—leaves; 2.94%—flowers), 1-octen-3-yl acetate (0.43%—leaves; 0.25%—flowers)	[47]
*A. rugosa*/ Republic of Korea	Leaves	Estragole (80.24%), linalool (4.23%), *dl*-limonene (3.50%), β-caryophyllene (2.39%), 1-octene-3-ol, *cis*-isopulegone, eugenol, β-bourbonene, β-elemene, β-humulene, β-cubebene, germacrene-D, bicyclogermacrene	[48]
*A. rugosa*/ Republic of Korea	Leaves	Limonene (47.09%), caryophyllene (17.01%), germacrene-D (14.31%), eugenol (4.72%), linalool (0.63%)	[49]
*A. rugosa*/ China	Flowers	Pulegone (34.1%), estragole (29.5%), menthan-3-one (16.7%), limonene (8.2%), 1-octen-3-ol, caryophyllene	[50]
*A. rugosa*/ China	Leaves	Menthan-3-one (48.8%), estragole (20.8%), limonene (7.7%), pulegone (0.4%), 1-octen-3-ol, caryophyllene	[50]
*A. rugosa*/ China	Leaves	Patchouli alcohol (45.70%), pogostone (11.92%), eugenol (8.29%), α-guaiaene (3.48%), β-elemene, β-caryophyllene, δ-cadinene(+)-, caryophyllene oxide	[51]
*A. rugosa*/ China	Uni-floral honey	Citral, citronellal, methyl octanoate-D, linalool oxide	[52]
*A. rugosa*/ China	Aerial parts	Methyl eugenol (50.51%), estragole (8.55%), eugenol (7.54%), D-limonene, linalool, pulegone, germacrene D	[53]
*A. rugosa*/ Germany	Aerial parts	Estragole (91.00%), caryophyllene oxide (0.89%), spathulenol (0.66%), linalool, 1-octen-3-yl acetate, eugenol, chavibetol, methyl eugenol, β-caryophyllene, germacrene D	[54]
*A. rugosa*/ China (Pulegone chemotype)	Leaves	(−)-Pulegone (59.01%), (−)-isomenthone (21.44%), (+)-limonene (9.04%), (+)-menthone (2.53%), germacrene D (1.29%), (+)-isopulegone (0.88%), caryophyllene (0.58%)	[55]
	Stems	(−)-Pulegone (36.27%), (−)-isomenthone (35.99%), (+)-limonene (22.27%), (+)-menthone (4.11%), γ-elemene (0.56%), caryophyllene (0.20%)	[55]
*A. rugosa*/ China (Estragole chemotype)	Leaves	Estragole (90.44%), methyl eugenol (6.89%), (+)-limonene (2.68%)	[55]
	Stems	Estragole (92.26%), (+)-limonene (5.83%), methyl eugenol (1.92%)	[55]
*A. foeniculum*/ Romania	Aerial parts	Estragole (94.89%), limonene (2.91%), caryophyllene (0.74%), eugenol, methyl isoeugenol, methyl eugenol, germacrene D	[26]
*A. foeniculum*/ Romania	Aerial parts	Chavicol, estragole, limonene, methyl eugenol, caryophyllene	[56]
*A. foeniculum*/ Romania	Leaves	Chavicol, estragole, eugenol	[56]
*A. foeniculum*/ Romania	Flowers	Estragole, methoxy-eugenol, limonene, methyl eugenol, eugenol	[56]
*A. foeniculum*/ Iran	Aerial parts	Estragole (97.2–98.1%), limonene (0.8–1.4%), β-caryophyllene (0.3–0.5%), 1-octen-3-ol, linalool, germacrene D, bicyclogermacrene, caryophyllene oxide	[57]
*A. foeniculum*/ Iran	Aerial parts	Estragole (94.00%), 1,8-cineole (3.33%), germacrene D (0.43%), octen-3-yl acetate, 1-octen-3-ol, 3-octanone	[58]
*A. foeniculum*/ Iran	Not mentioned	Estragole (74.90–92.73%), limonene, pulegone, spathulenol, caryophyllene oxide	[59]
*A. foeniculum*/ India	Aerial parts	Estragole (91.7%), limonene (3.9%), eugenol (0.9%), 1-octen-3-ol, linalool, β-caryophyllene, germacrene D, germacrene B, caryophyllene oxide	[60]
*A. scrophulariifolia*/ Belgium	Leaves	Isomenthone (49.7%), pulegone (19.8%), limonene (11.4%), 1-octen-3-ol, menthone, β-caryophyllene	[61]
*A. astromontana/‘Pink Pop’* Poland	Flowers and Leaves	Bornyl acetate (27.22–28.61%), linalool (23.76–24.65%), camphene (5.75–7.00%), *cis*-linalool oxide (4.70–5.12%), *trans*-linalool oxide (3.75–3.99%), eucalyptol (3.49–3.92%)	[2]
